# Transplantation of discarded livers: the complementary role of normothermic regional perfusion

**DOI:** 10.1038/s41467-021-24595-7

**Published:** 2021-07-22

**Authors:** Chris J. C. Johnston, Ahmed E. Sherif, Gabriel C. Oniscu

**Affiliations:** 1 Edinburgh Transplant Centre, Edinburgh, UK; 2 University of Edinburgh, Edinburgh, UK

**Keywords:** Biological therapy, Organ transplantation

**Arising from** Mergental et al. *Nature Communications* 10.1038/s41467-020-16251-3 (2020)

Mergental et al.^1^ present an important study that establishes objective parameters for the viability assessment of high-risk liver grafts. The study highlights that 71% of the retrieved and discarded liver grafts could be rescued and transplanted with initially good results, following a period of perfusion upon arrival at the transplant centre. However, an even larger number of livers are turned down based solely on pre-retrieval information, reaching 52% for the donors after circulatory death (DCD). As such normothermic regional perfusion (NRP) has a complementary role in further reducing the number of organs discarded by rescuing livers that are currently not even considered for retrieval.

Mergental et al.^1^ should be congratulated for pushing the boundaries in liver utilisation, but based on the data presented, at least 66 other livers could have been further evaluated and considered for transplantation if the VITTAL approach would have been used across more centres, to enable access to recipients of a suitable size and blood group, in addition to using other lower risk grafts. One essential question is whether the strategy proposed by the authors of this study is the appropriate one for all types of donors. This becomes clear when considering the reported incidence of biliary complications at one year: 30% in DCD compared with 8.3% in DBD and 18% in the overall comparative cohort. These patients required re-transplantation within 14 months. The authors acknowledge the failure of their strategy to mitigate against ischaemic cholangiopathy by stating: “it is clear that end-ischaemic NMP does not prevent the development of non-anastomotic biliary strictures in high-risk DCD organs”. Therefore we argue that discussion should consider the pivotal role of NRP which may be a better strategy in achieving this goal^2^. Whilst much of the current evidence supporting the benefit of NRP^2,3^ was not available at the time of VITTAL study conception, the results achieved with NRP whilst the VITTAL study was conducted reflect the extraordinary pace of change in the field of organ perfusion and preservation.

In contrast to the VITTAL approach where normothermic machine perfusion is initiated after a prolonged period of static cold storage (6.5–10.4 h in the current study), NRP re-establishes a circulation of oxygenated blood to organs in the donor, within minutes of circulatory arrest. This offers the opportunity to interrupt the cascade of biliary injury inflicted by a period of warm ischaemia followed by prolonged static cold storage and has been shown to markedly reduce the rate of ischaemic cholangiopathy, reported between 0 and 2% in a number of recently published clinical studies^2,3^. An alternative to the VITTAL approach is the initiation of NMP at the donor hospital^4^, which would further minimise the cold ischaemic time and maybe more directly comparable with NRP when undertaken for DCD donors. However, no study to date has demonstrated any significant reduction in ischaemic cholangiopathy over and above the ‘back-to-base’ model (consistently over 11%)^4,5^.

NRP is now established as routine clinical practice for multiorgan retrieval from DCD donors in two UK centres (Edinburgh and Cambridge)^3^ and in Spain^4^ and is mandatory for liver retrieval in France^6^. Our current protocol is to undertake organ retrieval after a period of two hours of in situ normothermic regional perfusion. An objective assessment of liver viability is undertaken during this period analysing multiple parameters similar to the ones used by Mergental et al.^1^, including sequential changes (measured every 30 min) in serum lactate and liver transaminases, glucose metabolism, pH normalisation and bile production.

Mergental et al.^1^ demonstrate an increased utilisation of marginal donor organs that have been retrieved but subsequently declined by all UK centres. However, a greater number of potentially transplantable organs exists within the cohort of DCD donors from whom liver retrieval is not even considered on the basis of donor history. Indeed, based on our experience over the last four years, we anticipate that an additional 30% of livers could potentially be rescued if donors are attended with NRP and undergo functional assessment.

Similar to the VITTAL experience, a greater number of organs could have been rescued if NRP would have been more widely available. Across the UK, from 1st April 2018–31st March 2019, 372 DCD donors proceeded with kidney (±pancreas) only donation^7^. Considering a 30% rescue rate based on the objective assessment criteria during NRP, it is conceivable that an additional 111 livers could have been offered for transplantation (in addition to the likely beneficial effects on kidneys and pancreases being retrieved from all of these donors^8–10^). We believe that objective in situ assessment of DCD liver viability facilitated by NRP (in addition to substantially improved outcomes in terms of function and ischaemic cholangiopathy) offers the opportunity to access an even larger number of transplantable livers that are currently not being retrieved.

The introduction of novel perfusion technologies will require a rethinking of transplant logistics. The approach proposed by Mergental et al.^1^ is less complex as it is undertaken at the base where all resources are available, but at present, shifting NMP implementation to donor hospital requires additional considerations such as time in donor theatre to prepare the liver (with potential vascular reconstructions) and placement on the machine. Furthermore, within a national organ retrieval system where a single team undertakes the retrieval of all abdominal organs (as is the case in the UK), additional training is required for the retrieval teams based in non-liver centres. In our experience, undertaking NRP at the donor hospital is less demanding logistically. NRP is delivered by the standard organ retrieval team with the addition of a single Advanced Specialist Practitioner in Organ Perfusion and Preservation (APOPS). In addition to logistics, technologies need further evaluation of cost-benefit. Whilst ex situ NMP is organ-specific and it comes at a high cost (£4000–20,000/case depending on device), abdominal NRP provides a benefit for all abdominal organs, at a lower cost. In our setting the costs of each NRP procedure is estimated at £4500 per case which includes the consumables, logistics and manpower.

NRP and NMP are complementary technologies that have much to offer in the current clinical context of liver transplantation. NMP is a landmark development in overcoming logistical challenges to liver transplantation in many clinical scenarios, in facilitating viability assessment of marginal grafts and more elective scheduling of liver transplants without the penalty of longer cold ischaemic times. Mergental et al.^1^ demonstrate this for DBD liver grafts but confirm the inability of NMP to ‘resuscitate’ DCD livers and reduce the risk of ischaemic cholangiopathy. NRP does offer this capability and allows for the identification of a large number of eminently transplantable DCD liver grafts that are not currently being retrieved. We are of the opinion that no liver should be discarded without assessment using NRP and NMP (alone or in combination) and the use of these two technologies is likely to markedly reduce the organ discard rates in the coming years. Future studies should be designed to assess the true benefit of these technologies with these clear goals in mind.

## Data Availability

The data that support the findings of this study are available from the corresponding author (G.C.O) upon reasonable request.
